# On the need for metaphysics in psychedelic therapy and research

**DOI:** 10.3389/fpsyg.2023.1128589

**Published:** 2023-03-31

**Authors:** Peter Sjöstedt-Hughes

**Affiliations:** ^1^Department of Sociology, Philosophy, & Anthropology, University of Exeter, Exeter, United Kingdom; ^2^Department of Psychology, University of Exeter, Exeter, United Kingdom

**Keywords:** metaphysics, mysticism, psychedelics, integration, philosophy, psychedelic-assisted psychotherapy, ontology, questionnaires

## Abstract

The essential proposal of this text is that psychedelic-induced metaphysical experiences should be integrated and evaluated with recourse to metaphysics. It will be argued that there is a potential extra benefit to patients in psychedelic-assisted therapy if they are provided with an optional, additional, and intelligible schema and discussion of metaphysical options at the integrative phase of the therapy. This schema (the “Metaphysics Matrix”) and a new Metaphysics Matrix Questionnaire (“MMQ”) stemming therefrom will be presented, the latter of which can also be used as an alternative or additional tool for quantitative measurement of psychedelic experience in trials. Metaphysics is not mysticism, despite some overlap; and certainly not all psychedelic experience is metaphysical or mystical—all three terms will be defined and contrasted. Thereafter psychedelic therapy will be presented and analysed in order to reveal the missing place for metaphysics. Metaphysics, with epistemology (theory of knowledge) and axiology (ethics and aesthetics), is a defining branch of Philosophy. Metaphysics, in contrast to mysticism, is considered to be based on argument rather than pure revelation. Thus, in psychedelic-assisted psychotherapy one sees here the potential bridge between reason-based philosophy and practical therapy—or, more broadly, with psychedelic-assisted psychotherapy there is the potential and mutually beneficial fusion of philosophy with practical science.

## Introduction

*Metaphysics should be used to integrate and understand psychedelic-induced metaphysical experiences*. That this proposition is near tautologically obvious yet not implemented in practice is the deficiency which this text seeks to remedy. Metaphysics is taken to be one of three core pillars of the discipline of Philosophy.^1^ Adorno (2001 [1965], p. 1) goes as far as to claim that “philosophy owes its existence to metaphysics.” Psychiatrist Humphry Osmond, in the 1957 paper in which he coined the word “psychedelic,”^2^ wrote that “perhaps most important: there are social, philosophical, and religious implications in the discoveries made by means of these agents” (1957, p. 432), paying heed therein to philosophers William James, Henri Bergson, John R. Smythies, and Immanuel Kant. There are indeed philosophical implications of psychedelic use,^3^ interwoven with the social and religious, and these implications can be used, I submit, to facilitate the integrative phase of psychedelic-assisted psychotherapy.

**Figure 1:**
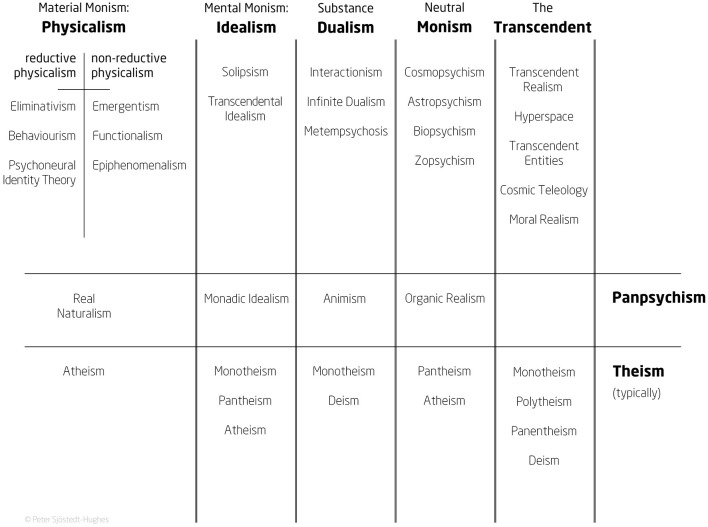
Metaphysics Matrix.

In this text we shall see how certain psychedelic experiences that seem to effect therapeutic outcomes in psychedelic-assisted psychotherapy can be viewed as intuitions or *experiences of* established metaphysical systems (outlined in Figure 1). For instance, experiencing the cosmos as sentient can be identified with the metaphysical system of Cosmopsychism (fourth column, first row of the Metaphysics Matrix). Enabling the recognition of such experiences as experiences of metaphysical systems through an additional and optional Metaphysics Integration strand within psychedelic-assisted psychotherapy could fortify, it will be proposed, the integration of a number of psychedelic experiences and thereby, it will be conjectured, lead to longer-term positive outcomes for certain patients—and life enrichment for others. Metaphysics is not mysticism (their differences are examined below); metaphysics is broader and its positions can be logically deliberated—as such metaphysics can *encompass* mystical experiences induced by psychedelic intake yet metaphysics can also *ground* those experiences in a manner that can be more intelligible, comprehensive, viable, and acceptable to participants than that which the framework of mysticism alone can offer. In brief, Metaphysics Integration could enhance psychedelic therapy.

Metaphysics concerns the fundamental nature of reality. It explores, as we shall see in the following section, issues such as the relation of mind to matter, to the cosmos, the nature of space, time, and causation, of self and identity, the possible and the eternal, of the nature of existence itself. Metaphysics—though it is neither mysticism nor physics—can perhaps be situated as a strict rational discipline between (or above, or below) both. Russell (1951 [1914], p. 1), in his essay on “Mysticism and Logic,” contends that “Metaphysics, or the attempt to conceive the world as a whole by means of thought, has been developed … by the union and conflict of two very different human impulses, the one urging men towards mysticism, the other urging them towards science.” Metaphysics is a rigorous subject that demonstrates its conclusions through logical argument rather than through empirical data, revelation, or intuition—*though certain metaphysical intuitions may trigger exploration of the arguments*. To give two examples: Bergson argued that Plato was catalysed in his dualistic metaphysical positions partly through initiation at the Mysteries (Bergson, 1935, p. 185–186; see also Inge, 1938, p. 392ff). Deleuze argued that the metaphysical position that is Spinozism could be attained *via* an “extraordinary conceptual apparatus” *or via* a “sudden illumination … a flash. Then it is as if one discovers that one is a Spinozist” (Deleuze, 1988 [1970], p. 129). Thus, metaphysics has both *intellectual* and *experiential* facets (Bossart, 1961; James, 1977 [1902], p. 373; Adorno, 2001 [1965], p. 137–145). Certain, but certainly not all, psychedelic experiences are metaphysical experiences. That such metaphysical experiences have accompanying metaphysical intellectualizations is a fact that can be utilised for psychedelic therapy and research.

Let us look more practically at the proposal: The protocol for psychedelic-assisted psychotherapy should, as its final phase—the *integrative phase* where the psychedelic-induced experience is reflected upon as regards its significance—include as an *optional* and *additional* element an intelligible metaphysical discourse based upon the Metaphysics Matrix (Figure 1). This matrix seeks to outline a reasonably comprehensive “menu” of metaphysical options, some of which may help experiencers to frame and thus make sense of, and give significance to, their experiences. In turn, it is conjectured that such additional metaphysical sense-making will increase the long-term benefits of psychedelic-assisted psychotherapy. This conjecture stems from two main seeds: First, there is compelling evidence that psychedelic-induced metaphysical experiences *per se* are a mechanism of positive psychological benefit (Roseman et al., 2018; Mollaahmetoglu et al., 2021; Rothberg et al., 2021; Yaden and Griffiths, 2021; Ko et al., 2022; McCulloch et al., 2022). Second is the thought that there may be less reason for the individual to come to reject the significance of such experiences as delusional once they realise that there may be more reason to bestow potential veridicality to the experience. The metaphysical positions of Platonism (a type of Universal Realism) and Spinozism (a type of Neutral Monism)—see Appendix 1: MMQ for a glossary of terms—for example, have, regardless of their truth or falsity, centuries of exacting arguments in their favour and thus cannot be dismissed flippantly. In this way, even a simple understanding of metaphysics may endow a lasting significance to the person who has undergone an associated metaphysical experience. At least, this is the conjecture, *a conjecture that can be tested*.

Psychedelic-assisted psychotherapy, though it has roots in the mid-20th century (Grof, 2008 [1980]), is still in its sapling stage. Much of it is based on psychotherapies and psychological theories that were developed without any explicit consideration of intensive metaphysical experience. That is to say that we are trying to repurpose a tool not designed for the matter at hand—using a hammer to correct grammar. From this perspective, it can be understood that integrating metaphysics within psychedelic-assisted psychotherapy is an adjustment that expedites the alignment of the therapy with its subject matter: metaphysics for metaphysical experience. Furthermore, it should be noted that integrating certain psychedelic experiences *via* metaphysics is not merely of potential benefit to those seeking help from mental ailments, but, outside the clinic, such metaphysical integration of psychedelic experience could be beneficial to “healthy” individuals and groups in terms of life enrichment.

**Figure 2:**
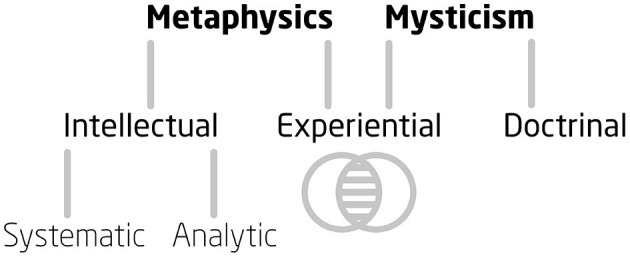
Metaphysics and Mysticism.

**Figure 3:**
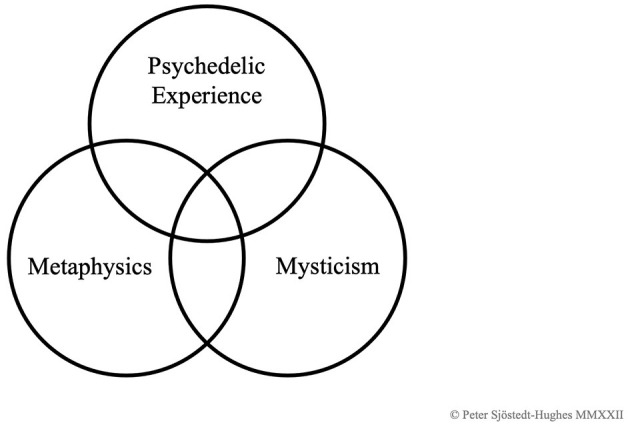
The PEMM Tristinction.

A secondary proposal is that this same Metaphysics Matrix can be used in clinical psychedelic trials to gauge the metaphysical nature of psychedelic experiences. A new questionnaire (Appendix 1), the Metaphysics Matrix Questionnaire (MMQ), can be used in addition to, or instead of, current questionnaires (see Herrmann et al., 2022) that are generally based on definitions of mystical experience alone (especially those based on Stace, 1960). But, as immediately concerns this paper, the MMQ can be used, as mentioned, as a *glossary* for the (capitalised) metaphysical positions referred to throughout. As we shall see, mystical experience—including “transpersonal experience” (Grof, 2009 [1975], p. 157–217) and “numinous experience” (Otto, 1926 [1917]; Spilka et al., 2003)—is not the same as metaphysical experience, even though there are overlaps (see Figures 2, 3). There are many metaphysical options that are not covered by mystical experience questionnaires—as will be explained in the sections below—which entails that the MMQ can augment the quantitative data in psychedelic trials and research, as well as in other research concerning metaphysical experience.^4^

In order to show how metaphysics may aid psychedelic-assisted psychotherapy, we shall examine what metaphysics is, how it maps on to particular psychedelic experiences and how it differs and has similarities to mysticism, what psychedelic-assisted psychotherapy is with a focus on integration, and how its deficiencies may be aided *via* augmentation with metaphysical integration conducing to, it is conjectured, longer-term mental health benefits. *In sum*, the proposal is to include Metaphysics Integration within psychedelic-assisted psychotherapy; the conjecture is that this will lead to longer-term therapeutic benefits.

## What is metaphysics?

The word “metaphysics” comes from the title of one of Aristotle's (384–322BC) texts (Aristotle, 2004), and the subject matter of metaphysics is thus related (yet developed) from that of the book. Aristotle's *Metaphysics* was named as such—by a later editor, possibly Andronicus of Rhodes, fl. c. 60BC (Marmodoro and Mayr, 2019)—because the texts that comprise the volume came *after* (*meta*) Aristotle's writings on physics (Aristotle, 2008). That bundle we now call Aristotle's book, *Physics*, however, also contains much of what we today would include within metaphysics (viz. change, infinity, time, an eternal cause). But, to step back, metaphysics, or “First Philosophy,” is, Aristotle writes, a “science whose remit is being *qua* being” (2004, p. 79 [Book Gamma, §1]). In other words, metaphysics' concern is the fundamental nature of existence itself. The Greek word for being, existence, is ó*ntos*, from which we get the term, “ontology,” which is at times used synonymously for metaphysics and at other times used as a subset of metaphysics. Yet if the latter, it is still the case that at “the heart of metaphysics is *ontology”* (Heil, 2021, p. 5). The way in which Aristotle analyses existence provides fundamental subject areas of metaphysics to this day, namely substance, causation, properties, relations, plurality and unity, species and genera, movement, identity, universals, particulars, categories, modality (potentiality, impossibility, actuality, necessity), form, space, time, matter, and divinity (the Prime Mover). Aristotle refers to and reviews the metaphysics of earlier thinkers such as Democritus (Physicalism), Pythagoras (mathematical Transcendent Realism), Parmenides (Neutral Monism), and especially his teacher Plato (Transcendent Realism, Substance Dualism)—it would therefore be an error to claim that metaphysics began with Aristotle: the discipline preceded the book.

In the 20th century, following the ascension of philosophies of language, logical behaviourism, and logical positivism, metaphysics became a derogatory term (Carr, 1987, p. 1; Beards, 2008, p. 10–19). It was a commonplace belief in philosophy (and other) departments that many, if not all, metaphysical issues—such as the relation of consciousness to matter, or the ontological status of time—could be reduced to, or explained away as, errors or meaningless expressions produced by the deceptive by-products of language. Though common, this negative evaluation of metaphysics was certainly not ubiquitous (see for instance Collingwood, 1940). Regardless, as the 21st century began to draw in, the limitations of these popular reductive philosophies—in the English-speaking world especially—were realised, and their own hidden metaphysical assumptions were brought to light. Consequently “there has been a quite remarkable revival of interest in metaphysics in Anglo-American philosophical circles during the last 30 years or so” (Beards, 2008, p. 10)—the so-called “metaphysical turn” (ibid., p. 11). This turn can be represented by the trajectory of the thought of Oxford philosopher A. J. Ayer, the most celebrated English advocate of logical positivism—the doctrine that a proposition is only meaningful if it is either empirically verifiable in principle or true by definition. In 1934, Ayer wrote an article named “Demonstration of the Impossibility of Metaphysics” declaring metaphysics annulled by these tenets of logical positivism (Ayer, 1934). But when asked in 1978 what the main shortcomings were of logical positivism, Ayer replied that “nearly all of it was false,”^5^ and in 1982 admitted that “Metaphysics is no longer a term of opprobrium” (Ayer, A. J., 1987 [1982], p. 140). Incidentally, in 1988 Ayer reported an intense psychedelic-like cosmic trip induced by a 4-min cardiac arrest brought on by choking on smoked salmon whilst already suffering pneumonia. The result of this single experience was his metaphysical supposition speculating that “death does not put an end to consciousness” (Ayer, 1990, p. 201).^6^ He died the following year.

Metaphysics is a demanding subject at university level. One might bifurcate the subject into two pathways. The first is the more traditional study of the work of established metaphysicians who often present an intellectual metaphysical *system*—figures such as the aforementioned Greeks, the neo-Platonists (Plotinus to Proclus), the “canonical seven philosophers” (Beaney, 2018) of the West (Descartes, Locke, Spinoza, Leibniz, Berkeley, Hume, and Kant), non-Western thinkers such as Rāmānuja, Lao Tzu, Nezahualcoyotl, or the more recent Nishida Kitaro (1870–1945) of the Kyoto School, et al. The second pathway is that of “analytic metaphysics” which is more concerned with the logic of separated-rather-than-systematic aspects such as the concepts of “substance,” “cause,” “disposition,” “identity,” “self,” “property,” “freedom,” “supervenience,” “possibility,” “space,” “time,” etc. (Carr, 1987; Kim and Sosa, 1999; Lowe, 2002; Marmodoro and Mayr, 2019; Heil, 2021).^7^ Both pathways inherit subject matter from Aristotle's *Metaphysics*, yet both develop and augment the discipline.

There is an (aforementioned) second sense in which metaphysics can be bifurcated, by which the previous bifurcation becomes the first branch of this more general one. There is what might be named *intellectual metaphysics* (divided into *systematic* and *analytic metaphysics*, as above), and there is *experiential metaphysics* (see Figure 2). Intellectual metaphysics is an abstract, *non-empirical* study—located as such between logic and physics. Experiential metaphysics is, on the other hand, as its name suggests, empirical. But it is not empirical in the normal sense of course: these doors, to employ Blake and Huxley's metaphor, are open. William James refers to experiential metaphysics when he writes that “in the nitrous oxide trance we have a genuine metaphysical revelation” (1977 [1902], p. 373; see also James, 1882, p. 206). To provide an example of the difference between intellectual and experiential metaphysics, respectively: one might give intellectual arguments for the feasibility of Pantheism (e.g. Sprigge, 1997, 2008), yet one may also, or instead, undergo an experience of feeling a beatific sentience that rolls through Nature. In the well-trodden words of Wordsworth:

“… And I have felt

A presence that disturbs me with the joy

Of elevated thoughts; a sense sublime

Of something far more deeply interfused,

Whose dwelling is the light of setting suns,

And the round ocean and the living air,

And the blue sky, and in the mind of man:

A motion and a spirit, that impels

All thinking things, all objects of all thought,

And rolls through all things. Therefore am I still

A lover of the meadows and the woods and mountains. ….” (Wordsworth, 1994, p. 207: The Tintern Abbey Ode)^8^

Physics and metaphysics both seek to understand the structure of reality. They may differ in their methods and content, but they are not of necessity in competition. A number of eminent physicists have been enamoured with metaphysics, such as Einstein, Schrödinger,^9^ and Heisenberg.^10^ Einstein was an ardent Spinozist, for instance, stating that “Spinoza is the greatest of modern philosophers, because he is the first philosopher who deals with the soul and the body as one…” (stated in an interview for Viereck, 1930, p. 373). And it is here where physics and metaphysics essentially differ: metaphysics is very much concerned with *mind* and its relation to body, or to physicality and the cosmos as a whole—whereas physics has little to no concern for mind. The reasons for this distinction are historical (see Whitehead, 1935 [1925]; Collingwood, 1945) and not relevant here. What is relevant is that this mind-matter problem is a core issue in metaphysics and one that relates intellectual to experiential metaphysics. We can move down within metaphysics, into ontology, into the issue of *substance*—that which “sub-stands” under all, that which is the fundamental stuff (or process)^11^ of reality. The question is what substance *is*. Is it matter (Physicalism)? Is it mind (Idealism)? Is it both matter and mind (Substance Dualism)? Is it something that incorporates matter and mind (Neutral Monism)? Is it something that transcends matter and mind (Transcendent Realism)? Are there further options? All known options have numerous arguments in their favour, and innumerable criticisms. There is no default, standard option. Even Physicalism is, ironically, a metaphysical position and should not be adopted without due caution. One cannot avoid metaphysics. As mathematician and philosopher Alfred North Whitehead said, “If you don't go into metaphysics, you assume an uncritical metaphysics” (quoted in Petek, 2022, p. 43). One can only judge a viewpoint and its experience to be *delusional* if one knows what reality is^12^—and there is no agreement. We do not know the solution to the “hard problem of consciousness,” *the mind-matter problem*—what the *relation* is between mind and matter (Chalmers, 1995; Kim, 2005, p. 7–31). More specifically, to use philosophic parlance, we do not know the necessary and sufficient conditions for mind. We should therefore keep our minds open to a range of metaphysical positions.

These aforementioned metaphysical, ontological positions have been placed as the columns of the Metaphysics Matrix (Figure 1). These positions, defined here *via* the MMQ (Appendix 1), are capable of being understood both intellectually and experientially. Indeed, it is the intention here to relate psychedelic- (and otherwise-) induced metaphysical experiences (experiential metaphysics) to their associated intellectually cognized metaphysics so to gain a greater understanding of people's experience and, in so doing, to attain the possibility of bestowing more significance to the experience. If it is the case that it is metaphysical experiences that provide the mechanism for positive outcomes in psychedelic therapy (see below), then—to reiterate—it is the conjecture here that the ability to frame the experience metaphysically will yield longer-term positive outcomes because one is less likely later to reject an experience that correlates to an intellectual position supported by reason through an established, eminent lineage of rigorous debate.

The Metaphysics Matrix is non-exhaustive—there are many other options not there presented. This is because it is designed for practicality rather than exhaustivity. It contains the main strands of metaphysics, in the core sense of ontology, as five columns and two rows. Panpsychism (that minds are ubiquitous in Nature, from the human to the subatomic) takes its own row in order to traverse varieties physicalist, idealist, dualist, and neutral monist. As Skrbina (2007, p. 2) argues, panpsychism “is a meta-theory of mind.” Popper and Eccles (1985 [1977], p. 67–71), Strawson and Freeman (2006), and Strawson (2016) for example, classify (with qualifications) Panpsychism as a Physicalist (or, Material Monist) theory; Schopenhauer (1969 [1818]) and Leibniz (1991 [1686, 1714]) offer an Idealist (Mental Monist) version of Panpsychism [Popper and Eccles (ibid., p. 68) quips that “Schopenhauer is a Kantian who has turned panpsychist”]; Spinoza offers a Neutral Monist Panpsychism (1988 [1677], p. 457–458: *Ethics*, IIP13s), which despite its age is still considered by some leading metaphysicians as the most feasible variety. Heil (2021, p. 130), for example, posits that “for Spinoza, consciousness is an attribute pervading the universe, something like a field suffusing spacetime. ... [M]inds might be local concentrations ... the most plausible form of panpsychism.”

Consequently, it is inadequate to simply differentiate Panpsychism in opposition to Physicalism, Dualism, etc. In a recent empirical study reported in a paper named “Psychedelics alter metaphysical beliefs” by Timmermann et al. (2021), it was attested that psychedelics tend to shift people's beliefs away from Physicalism (“hard materialism”) to Panpsychism. Though this may be suggestive of the truth, it should be treated with a little caution. Firstly because, as noted, Panpsychism is a meta-theory and so can be seen as a type of Physicalism, etc. Secondly, the thirteen-item Metaphysical Beliefs Questionnaire, developed for the study, defined Panpsychism in a way that could also be mistaken for Idealism. There are also certain omissions in the study, notably that of Neutral Monism, and its associated doctrines of Cosmopsychism or Pantheism (God is Nature)—metaphysical views considered by many as central to a peak psychedelic experience (Stace, 1960, p. 207–218; Shanon, 2010 [2002], p. 163ff; Lundborg, 2014, p. 87ff). Though anecdotal, there are also documented cases of psychedelic experience bolstering the metaphysical belief in Physicalism (Langlitz, 2013, p. 204–241).^13^ Nonetheless, the general empirical finding that psychedelics have a tendency to shift metaphysical beliefs also implicitly suggests that more focus needs to be placed on metaphysics in psychedelic-assisted psychotherapy.

Theistic views also cut across the ontological columns of the Metaphysics Matrix as a row so to allow for beliefs in divinity without need for specific beliefs in any particular mind-matter ontology. Theism was a part of Aristotle's *Metaphysics* (Book Lambda) wherein he argued for a (non-religious) god (the “Uncaused Cause,” or the “Prime Mover”). Whitehead (1935 [1925], p. 249) notes that “Aristotle found it necessary to complete his metaphysics by the introduction of a Prime Mover—God. ... [He] was entirely dispassionate; and he is the last European metaphysician of first-rate importance for whom this claim can be made. After Aristotle, ethical and religious interests began to influence metaphysical conclusions.”

It is not the ambit (or possibility) of this paper to explicate the various multicultural, metaphysical positions outlined in the Matrix and its MMQ. This will be the ambition of a separate forthcoming handbook for use by practitioners, patients, and various other psychonauts. Here, it is sufficient to describe what metaphysics is, how it relates to psychedelic experience, and how it differs from, and has similarities with, mystical experience. The notion of “mystical experience” has dominated psychedelic surveys and trials hitherto, so let us look at it.

## What is mystical experience?

To ask for a definition of “mystical experience” is to immediately become embroiled within three controversies. The first is simply that the modern definition is ambiguous and thus the scene of embattled conceptual nets (Spilka et al., 2003, p. 299). The second is that it is disputed whether there exists one qualitatively identical, transcultural, perennial, common core mystical experience to be defined. This “perrenialism,” or “perennial philosophy” (*philosophia perennis*) was coined and developed by Roman Catholic (Augustinian) theologian and Renaissance (neo-) Platonist Agostino Steuco in 1540 (Schmitt, 1966). Against this lineage of perennialism that maintains that there is such a common core mystical experience (Leuba, 1925; Stace, 1960; Staal, 1975; James, 1977 [1902]; Huxley, 2004 [1954/1956]) is opposed the “contextualism” that asserts (Katz, 1978, 1983) that personal and cultural contexts condition not merely the interpretation and report of an experience but the very experience itself. To simplify: Perennialism claims that mystical experience *deconditions* one from culture; contextualism claims that mystical experiences are *conditioned by* culture. There is a third way of course—Ann Taves, for example, argues that though culture determines much of a psychedelic-induced mystical experience, a high-enough dose of a potent psychedelic drug is also a significant causal factor: “the differential effects of both types and doses of psychedelics on participants clearly establish that the subjects' experiences are not simply the result of these [cultural] factors” (Taves, 2020, p. 679). Set and setting play an important role, but drug and dose play one too. Here is not the place to explore this debate (see Baier, 2017), though it obviously has potential ramifications for the objectivity of certain psychedelic phenomenological studies. It should further be noted that perennialism can be divided into those who believe the common core mystical experience is veridical, and those who believe that, though it is a transcultural, human experience, it is nonetheless a delusion. The third controversy, that will also be avoided, is the question as to whether psychedelic drugs, rather than seasoned religious practice, can occasion genuine mystical experience (if there is such a thing). Zaehner (1961 [1957], 1972) and Suzuki (1971), for instance, argued they cannot (see Odin, 2022 on the latter).

We will have to enter the first controversy, however, to loosely define and thereby make intelligible for discussion “mystical experience,” so that we can thereafter compare and contrast it to metaphysics. We begin with the etymology of “mystical experience,” reducible to “mystic.”

From the Greek root *myo*, meaning “to close” (the eyes), we get *mys-tes*, meaning an “initiate” into secret rites (Oxford Classical Dictionary), a “mystic.” This relates to the Mystery sects of ancient Greece, the most established of which were the Eleusinian Mysteries (Kerényi, 1967). The first known reference to “mystic” (*mys-tes*) comes from the philosopher Heraclitus (fl. c. 500BC) (frg. B15 DK; ibid.). Plato also speaks of the Mysteries, wanting himself to be counted a mystic (*Phaedo*: Plato, 2002, 69c–d), and we glimpse here Bergson's and Russell's contention that mysticism had a catalytic effect upon metaphysics. Aristotle also spoke of the Mysteries, writing that (frg. 15) “initiation did not teach (*matheîn ti*) but rather conveyed an experience (*pathein ti*)” (Oxford Classical Dictionary), reflecting another relation between metaphysics and mysticism, respectively. Before the Mysteries were closed down after two millennia late in the fourth century by the Christian Roman Emperor Theodosius I, the pagan philosopher Plotinus (AD204/5–270) had developed a monistic mystical metaphysics that would found a school (Neoplatonism) and that would have much influence upon Christianity and thus the Western understanding of “mystical experience” (Spilka et al., 2003; Gertz, 2022, p. 299 (referencing Albrecht Ritschl); Katz, 1978, p. 41; Leuba, 1925, p. 305). The central tenet of Plotinus' metaphysical system is “The One.” Plotinus arrives at this tenet through means both intellectual (*via* Plato and Philo) and experiential. In the *Enneads*, Plotinus writes that:

“The man who obtains the vision becomes, as it were, another being. He ceases to be himself, retains nothing of himself. Absorbed in the beyond he is one with it, like a center coincident with another center. While the centers coincide, they are one. They become two only when they separate. It is in this sense that we can speak of The One as something separate.

This, doubtless, is what is back of the injunction of the mystery religions which prohibit revelation to the uninitiated. The divine is not expressible, so the initiate is forbidden to speak of it to anyone who has not been fortunate enough to have beheld it himself. The vision, in any case, did not imply duality; the man who saw was identical with what he saw. Hence he did not ‘see' it but rather was ‘oned' with it.” (*Ennead* VI, 9 [9], §§10–11: O'Brien, 1964, p. 87)

This sense of unity of the self with a greater existence thus lies at the heart of the current understanding of mysticism. Underhill (1914, p. 3) writes that “Mysticism is the art of Union with Reality.” In James H. Leuba's 1925 tome, *The Psychology of Religious Mysticism*—wherein incidentally there is a chapter on mystical ecstasy produced by drugs (viz. alcohol, mescaline, hashish, ether, and nitrous oxide)—Leuba (1925, p. 1) defines “mystical” thus: “[The] term ‘mystical' … will mean for us any experience taken by the experiencer to be a contact (not through the senses, but ‘immediate,' ‘intuitive') or union of the self with a larger-than-self, be it called the World-Spirit, God, the Absolute, or otherwise.” This definition takes inspiration from Plotinian philosophy, but also from the then-concurrent milieu of Hegelian Idealism (“World-Spirit,” *Weltgeist*) and its offshoot, Absolute Idealism (“the Absolute”) (see Robbins, 1982). Leuba speculates that Plotinus was also inspired by Vedanta philosophy (1925, p. 305)—regardless of the scarcely substantiated truth of which, the influence and fusion of such Eastern thought upon Western notions of mysticism cannot be doubted (see Lenson, 1995, p. 144ff: “Acid Metaphysics”). Philosopher and psychologist James (1977 [1902], p. 404) too epitomises mystical experience in likewise fashion: “In mystic states we both become one with the Absolute and we become aware of our oneness.” Carpenter (1892) named such a state “Cosmic Consciousness”;^14^ Freud (2002 [1930], p. 3ff), borrowing from personal letters from Romain Rolland, referred to this unbounded feeling as the “Oceanic” (Rolland used it originally in reference to “the flash of Spinoza”—see Sjöstedt-Hughes, 2022). In terms of this paper's intention, it can be noted here that though mystical experience appears intuitive rather than intellectual, *the intuition is framed from the historical start in intellectual metaphysics* for intelligibility and added *significance*. The neo-Cambridge Platonist W. R. Inge laments, against the dominantly psychological study of mysticism inaugurated in the 20th century, that “mysticism is essentially ontological; the contemplative cares nothing for states of consciousness. His business is with the ultimately real.” (1938, p. 388). There is then an overlap between mystical and metaphysical experience: “Union” (of Self and Greater) can be intellectualised through a number of metaphysical systems such as Transcendent Realism and Idealism. Wider still, if mystical experience is essentially and concisely defined as believed “direct experience of ultimate reality” (Carmody and Carmody, 1996, p. 10), and if metaphysics is defined as concerning “the fundamental structure of reality” (Lowe, 2002, p. 2–3), then metaphysics concerns itself with the ultimate reality that mystics claim to experience, as well as aspects of reality beyond mystical experience (see Figure 3). Thus psychedelic-assisted psychotherapy might attain more meaningful and significant outcomes were it to employ metaphysics *to frame* such metaphysical experiences.

William James, in his 1902 book *The Varieties of Religious Experience*, catalysed the academic interest into mystical states (Inge, 1938, p. 387), and furthermore advanced the view that such states can be occasioned through chemical agents — though there were earlier exponents of this view: it was Benjamin Paul Blood who pushed James into this very idea with his unusual little book of 1874: *The Anaesthetic Revelation and the Gist of Philosophy* (Blood, 2020 [1874]). Almost a decade after *The Varieties*, William James put forward an explicitly Fechnerian theory of mystical consciousness, related to Cosmopsychism and psychophysics which he fused with the philosophy of Hegel and Bergson (James, 1909, 1910). However, mysticism is obviously understood in ways other than this, and in ways other than mere union with reality, or reality as union. It is beyond the ambit of this text to elaborate on all of these varying definitions and classifications of mystical experience. But let us enumerate certain criteria (not exhaustive) by which “mystical experience” has been understood, so to provide a little more overview of what “mystical experience” is taken to be, and to provide references for further exploration.

- James (1977 [1902], p. 367ff)
  1. Ineffability
  2. Noetic quality
  3. Transiency
  4. Passivity

- Underhill (1911, p. 78ff)
  1. Activity and Practicality
  2. Transcendent intentionality
  3. Love of The One
  4. Unitive State

- Russell (1951 [1914], p. 12ff)
  1. Intuitive (not Rational)
  2. Unitive (not Plural)^15^
  3. The Unreality of Time
  4. Beyond Good and Evil

- Otto (1926 [1917], p. 12–41)^16^
  1. Awefulness
  2. Overpoweringness
  3. Energy
  4. The wholly other

- Stace (1960, p. 79ff)
  A. Introvertive and Extrovertive Mysticism
  1. The Unitary consciousness; The One, the Void, Pure Consciousness
  2. The immanence of The One in all things
  3. Sense of objectivity or reality
  4. Blessedness, peace, etc.
  5. Feeling of the holy, sacred, or divine
  6. Paradoxicality
  7. Alleged by mystics to be ineffable
  8. Non-spatial, non-temporal (introvertive only)

- Zaehner (1961 [1957], p. 93ff)
  1. Transcendence of Space (thus Unity)
  2. Transcendence of Time (thus Unity)
  3. Contraction into The One (Being not Becoming)
  – Peaceful, Joyful
  – Beyond Good and Evil
  6. The Love of God (Beyond The One)
  7. A. Zaehner also distinguished Nature Mysticism, Soul Mysticism, and Theistic Mysticism.

Stace's criteria—*via* Pahnke, Richards, Hood, et al.—were those that informed the most common mystical experience questionnaires, viz. the Mystical Experience Questionnaire (MEQ) (Pahnke, 1963), and the Hood Mysticism Scale (the “M-Scale”: Hood, 1975) that are today used in psychedelic trials (also in use are the Hallucinogen Rating Scale, and that of the Five Dimensions Altered State of Consciousness—see Herrmann et al., 2022 for a recent overview of all scales used in psychedelic research). Data derived in this manner is obviously limited and abstract not only because psychedelic experience need not be “mystical,” but also because the definition of “mystical” could be expanded to include other criteria observed above (e.g., transcending good and evil), and even these are non-exhaustive. With regard to psychedelic-assisted psychotherapy, as we shall see, speaking about mystical experience *per se* will not be sufficient to provide a meaningful explanation of the significance of such experience to a person, for the simple reason that mystical experience is the phenomenon to be explained—*mystical experience is the explanandum rather than the explanation*. It is metaphysics that is the means of explanation, the *explanans* of the mystical *explanandum*.

One can distinguish an explanation of a psychedelic experience within therapy from an academic explanation. With regard to the latter, it should be noted that simply reducing mystical experience to neural correlates is *not a sufficient explanation* because the so-called neural correlates of consciousness (Koch et al., 2016) *present rather than solve* the mind-matter problem. As philosopher of mind Jaegwon Kim (2005, p. 13) makes clear, following James, “Making a running list of psychoneural correlations does not come anywhere near gaining an explanatory insight into why there are such correlations.” Thus, when Carhart-Harris et al. (2018, p. 549) claim “Our work on the neural correlates of ‘ego-dissolution' may be considered part of a progressive initiative to demystify the psychedelic experience … a candidate neural correlate of the unitive experience,” they are only mystifying themselves. Though neural correlates are *part of* an explanation of such experience, they cannot be a *sufficient* explanation, because the relation between the phenomenology and the physiology is left unexplained. Again, the mind-matter problem keeps the metaphysical options open. Making aware the question of this basic openness would itself be part of the proposed Metaphysics Integration phase, outlined below.

We can now see the difference between metaphysics and mysticism; the two should not be conflated despite certain similarities (see Figure 2). Metaphysics is more comprehensive than mysticism, and as such provides secular frameworks *in which* to understand the significance of “mystical experiences.” But metaphysics also provides frameworks for other forms of exceptional experience that are often excluded from mystical criteria. The Penzance-born “chemical philosopher” Humphry Davy, for instance, after inhaling 200 pints of nitrous oxide exclaimed a revelation of Idealism (“Nothing exists but thoughts!”—Davy, 1800, p. 490). In the following section we shall look at psychedelic-induced metaphysical experiences, matching them to the Metaphysics Matrix, in order to show how the Matrix can be used. Metaphysics is an ongoing, active discipline that can be fruitful to the emerging field of psychedelic studies, in terms of therapy as well as individual and cultural enrichment. In what follows, we shall also observe how metaphysics may be applied specifically to the integrative phase of psychedelic-assisted psychotherapy so to potentially increase long-term mental health benefits to participants.

## Psychedelic-induced metaphysical experiences

Humphry Osmond coined and defined “psychedelic” as “mind-manifesting” (1957, p. 429), not “brain-manifesting,” and thus the term “psychedelic” should be determined by drug-induced (“agents”) *phenomenology* rather than physiology. Moreover, Osmond was explicitly against defining the term by purely neuropharmacological determinants (ibid., p. 428)^17^ so we cannot restrict “psychedelics” to agents that that act primarily on the serotonin receptors. Osmond included nitrous oxide in his paper, in reference to William James, and it would be implausible to exclude the potent *Salvia divinorum* from the categorisation of psychedelics.

There is thus a long range of chemicals that may be classified as psychedelics (see Shulgin and Shulgin, 2019 [1991], 2020 [1997]), and an even longer range of experiences that may be classified as psychedelic experiences. Even a single type of chemical can elicit a seemingly infinite variety of experiences, though psychedelic typologies have been attempted (Lewin, 1998 [1924]; Masters and Houston, 2000 [1966]; Grof, 2009 [1975]; Shanon, 2010 [2002]). It is, consequently, manifest that not all psychedelic experiences are either metaphysical or mystical. Even the popular writer on psychedelics, mysticism, and Eastern thought, Alan Watts, wrote that: “my first experiment with LSD-25 was not mystical. It was an intensely interesting aesthetic and intellectual experience” (Watts, 2013, p. 98). Psychedelic experience can elicit laughter, unusual or intensified bodily feelings, they can be used to sharpen the senses and endow a person with enhanced aptitude for courage and stamina (MacCreagh, 2016), for hunting or fighting, or they can be used with the intention of cursing or healing others through sorcery or *via* the invocation of beings—as documented by Yanomami shaman, Davi Kopenawa (Kopenawa and Albert, 2013, p. 113–151). Likewise, there are aspects of mystical experience that bear little relation to metaphysics or psychedelics, especially those experiences tied to certain religious doctrines or denominations, such as the Christological visions of the counter-Reformationist Carmelites, such as St Teresa of Ávila (1515–1582) and St John of the Cross (1542–1591). And thirdly, there are aspects of intellectual and experiential metaphysics that bear little relation to psychedelic or mystical experience, such as the dry study of causation, identity, supervenience, etc. There is, however, an area of overlap of all three—all of which is illustrated in the Venn diagram that is Figure 3, the “PEMM Tristinction”: Psychedelic Experience, Metaphysics, and Mysticism. For this paper, we are concerned with the space of overlap between psychedelic and metaphysical experience, which also, as illustrated, overlaps with some mystical experience.

It should be reiterated that many of the items listed in the mysticism scales and in the mystical experience criteria lists, both presented above, are items that are *assimilated within* the metaphysical positions outlined in the Metaphysics Matrix. For instance, the felt Union with reality (and its derivatives such as “nature-connectedness” [see Inge's “Nature Mysticism” (1938, p. 396); or the Watts Connectedness Scale (Watts et al., 2022)], can be assimilated and thus made meaningful within Neutral Monism, and relatedly to varieties of Panpsychism. Yet we must be cautious of generalizing: the word “union” can have various meanings—as Katz (1978) made clear: e.g., Jewish *devekuth* and Buddhist *nirvana* appear to be different experiences yet both terms can be translated or conflated with the single word “union” (Katz, 1978, p. 29ff; see also Jylkkä, 2022). Further still, “Neutral Monism” has a variety of meanings or variants. The specificity of every term must be fathomed by qualitative discussion between the experiencer and the integrator—this is the core component of the Metaphysics Integration, outlined below. The possibility of assimilating psychedelic-induced metaphysical experiences into metaphysical positions is possible by definition, i.e., not impossible.

Further still, the types of psychedelic-occasioned mystical experience that show most correlation to, mediation of, and prediction of, therapeutic effect are those experiences that are also classified as metaphysical experiences and thus are experiences that can be *further explored and understood* through Metaphysics Integration (mysticism by definition stays at the stage of mystery). For instance, Roseman et al. (2018) found experiencing “oceanic boundlessness” predicted a decrease in depressive symptoms. The very term, as we have seen, derives, *via* Freud and Rolland, to Spinoza's variety of Neutral Monism (Sjöstedt-Hughes, 2022). What better method of integrating such a metaphysical experience than through discussion of the very metaphysics that spawned its term? Certainly, I submit, integration of such experiences without any recourse to metaphysics would be inadequate—with the qualification that Metaphysics Integration *alone* is certainly inadequate as well. Developed and established psychotherapeutic (or logotherapeutic)^18^ methods that counsel a participant are no doubt necessary elements of psychedelic integration. Hence the proposal that Metaphysics Integration be an *additional* part of psychedelic-assisted psychotherapy. It should also be *optional*, to offer agency to the patient, and because a patient may not have undergone a psychedelic-induced metaphysical experience. Another point of metaphysical significance is that if it is the phenomenology rather than the physiology that is primarily therapeutically effective here (contra Olson, 2020), therewith comes an implicit assertion of *mental causation*. Mental causation is problematic for a number of metaphysical positions (such as Dualism and Physicalism—see Kim, 2005), and is related to the long-running metaphysical controversy between Free Will, Determinism, and Fatalism (Lucas, 1970). But here is not the place to explore the hitherto-unregistered yet potentially serious ramifications of this issue for psychedelic research and therapy.

To further outline how such an additional and optional Metaphysics Integration scheme may be run, let us give a few more examples of how psychedelic-induced metaphysical experiences can be assimilated within metaphysical positions, many of which overlap with one another. The discreteness of terms often masks the interrelation of realities.

In *The Antipodes of the Mind: Charting the Phenomenology of the Ayahuasca Experience*, Benny Shanon (2010 [2002], p. 163) writes that, “Overall, Ayahuasca induces a comprehensive metaphysical view of things,” continuing to aver that the “experience forced ontology on me” (ibid., p. 165). But of which type? Shanon: “I would characterise it as idealistic monism with pantheistic overtones” (ibid., p. 163). As well as referencing Plato, Plotinus, and Hegel, Shanon makes reference to certain Hindu philosophies in this respect. Shanon also points out that cognitive psychology is inadequate to deal with such experience (ibid., p. 380). We can locate such an experience in the Metaphysics Matrix (second column, third row), and note that Pantheism (God is the Universe) is a rather commonly reported psychedelic-induced experience that encompasses and provides an explanatory framework for other experiences such as nature-connectedness, union, timelessness, ego-loss, etc. A classic exclamation of such an experience is given by Alan Watts as “peculiar states of consciousness in which the individual discovers himself to be one continuous process with God, with the Universe” Watts (1968 [1962], p. 74). Pantheism was coined by Joseph Raphson in 1697 in reference to Spinoza's metaphysical system (Spinoza, 1985/1988). Spinozism is more a Neutral (than Idealist) Monism (i.e., mind and matter are fundamentally identical because they are expressions of a more ultimate reality: God/Nature) and is one that can be used to discuss relevant psychedelic-induced experiences. Albert Hofmann sympathised and entertained such a view when he used as an epigraph for the final chapter of his book, *LSD: My Problem Child*, a line from Goethe referencing Spinoza: “What more can a person gain in life than that God-Nature reveals Himself to him?” (Hofmann, 2009 [1979], p. 197; see also Sjöstedt-Hughes, 2022 for a comparative analysis between Spinozan ontology and psychedelic, particularly 5-MeO-DMT, phenomenology). In his chapter, Hofmann is incidentally in alignment with the purpose of this paper when he writes that: “[A] type of ‘metamedicine,' ‘metapsychology,'… is beginning to call upon the metaphysical element in people … and to make this element a basic healing principle in therapeutic practice.” (ibid., p. 206).

Pantheism is related to Panpsychism (that minds are ubiquitous in Nature), and we see such a view expressed throughout the psychedelic literature. The aforementioned Timmermann et al. (2021) study indicated a general metaphysical shift from Physicalism to Panpsychism *via* psychedelics, thereby also showing the commonality of the position in psychedelic experience. In 1957, Richard H. Ward described a panpsychological experience induced by 100 micrograms of LSD:

“This insight into the hidden nature of things, which in itself swept away time and distance and left me with a new understanding the authenticity of which I could not doubt, was indescribably thrilling: this was really to know, really to feel, and to transcend the mean limitations of our ordinary ways of perceiving …. I realized that the whole universe is made-up of things which have their own natures, relationships, significances, and that in some universal scale each thing has its proper place and even its proper degree of awareness, whether we call it animate or inanimate.” (Ward, 1957, p. 85–86)

A variant of Panpsychism is Animism, an essential stance of Amerindian cultures related to the use of psychedelic substances such as ayahuasca (see Shanon, 2010 [2002]; Kopenawa and Albert, 2013; Luna, 2020, p. 167f). The relation between Animism and Panpsychism is an interesting one to unravel, as is the fact that experiencing the seemingly inner life of things is an experience common to both Western and Amerindian cultures, and beyond (e.g., in Shintoism—see Yoneyama, 2017).

With regard to Substance Dualism (that the soul is separate from the body), the reports are manifold. Reports of psychedelic-induced past lives—metempsychosis (Grof, 2009 [1975])—imply a Substance Dualism as a soul must, by this doctrine, survive one body to enter the another. Reports of “astral projection” also entail such a dualism as the soul is supposed to leave the body (Foss, 1973). Moreover, the vast literature of reports on encountering sentient beings (Strassman, 2001; Gallimore, 2019; Michael et al., 2021) *generally* implies that those supposed sentiences—with their own subjective perspectives—have an existence without a physical subvenient base. This would in turn imply a type of substance dualism as the sentience is, as it were, free floating. Though Substance Dualism is not a currently popular viewpoint in philosophy or science today, it does have its forceful proponents (e.g., Popper and Eccles, 1985 [1977], p. 36–99; Feser, 2010, p. 19–48), and can be intelligently discussed without necessary reduction to mere religious faith or intuition alone. For certain Plotinian purists who confine “mysticism” with union—understood as such through a Monism—experiences of Dualism are ruled out as mystical *a priori*. Inge (1938, p. 404), for example, contends that “Metaphysical dualism is inconsistent with mystical philosophy.” This issue is irrelevant to our purpose here because we are looking at metaphysical rather than purely mystical experiences with regard to integration. But it again reveals that psychedelic-assisted psychotherapy will gain more advantage by extending its scope beyond mysticism to metaphysics.

With regard to Idealism, we have seen its evocation *via* nitrous oxide for Humphry Davy. William James also arrived at Idealism *via* nitrous oxide, Idealism of the Hegelian variety. In the extensive endnote to his 1882 paper, “On Some Hegelisms,” he writes:

“I have made some observations on the effects of nitrous-oxide-gas-intoxication which have made me understand better than ever before both the strength and the weakness of Hegel's philosophy. … [The] keynote of the experience is the tremendously exciting sense of an *intense metaphysical illumination*. … [Its] first result was to make peal through me with unutterable power the conviction that Hegelism was true after all, and that the deepest convictions of my intellect hitherto were wrong. … [U]nbroken continuity is of the essence of being; and … we are literally in the midst of *an infinite*, to perceive the existence of which is the utmost we can attain.” (James, 1882, p. 206)

We have looked briefly at how certain psychedelic-induced experiences can be framed and more adequately understood through certain metaphysical systems: Neutral Monism, Pantheism, Panpsychism, Animism, Substance Dualism, and Idealism. But it should be noted that many elements of the psychedelic experience are not as systematic, not as totalizing. There are many interesting non-systematic experiences that can also be meaningfully understood through metaphysics. I refer to experiences such as the contraction or protraction of timerate, or of the specious present; of timelessness (“the Eternal”); of lost memories (in relation to the ontological status of the past); of spacelessness or of a space of other than three dimensions; of the platitudinous “ego-loss” (assimilated within Neutral Monism, etc.); of nature-connectedness (related to Panpsychism, Pantheism, etc.); of other realms (in relation to Transcendent Realism, and to “Possible World” modal realities); to supreme aesthetic experiences; to the seeming diffusion and proliferation of the mind; to the amplification of the feelings and emotions; to the divine (following Aristotle's legacy, we consider non-denominational, natural theology as one part of metaphysics). All such experiences, and others, can be discussed using the discourse of metaphysics.

If certain such metaphysical experiences promote therapeutic benefits it would behove us to endeavour to accentuate through exploration these experiences in the integrative phase of psychedelic-assisted psychotherapy (and perhaps, in time, beyond that).

## Psychedelic-assisted psychotherapy

Gorman et al. (2021) have defined psychedelic-assisted psychotherapy thus: “the administration of a psychedelic in the context of a psychotherapeutic environment and relationship, with the therapist providing psychological support and in some cases specific intervention designed to align with the psychedelic experience and promote change in the target diagnosis” (p. 3). It is, and has been, used to attempt to treat diagnoses of posttraumatic stress disorder, depression (major depressive disorder, treatment-resistant depression), addiction, pain, anxiety, obsessive compulsive disorder, schizophrenia, end-of-life care (thanatophobia, etc.), and other afflictions deemed harmful (ibid.; Grof, 2008 [1980]; Morgan et al., 2017; Garcia-Romeu and Richards, 2018).

The therapeutic use of psychedelics appears to have a long history in ancient and indigenous cultures (Osmond, 1957, p. 419; Schultes et al., 1998 [1979]; Escohotado, 1999 [1996]; Rinella, 2012). In the clinical sphere of the West, therapeutic use of psychedelics, at first LSD—synthesised in 1938; taken first by Albert Hofmann in 1943 (Hofmann, 2009 [1979])—began in the early 1950s (Grof, 2008 [1980], p. 26; Garcia-Romeu and Richards, 2018, p. 292). From the 1950s to the start of the 1970s (when prohibition of such substances was implemented by the UN), there were two general tendencies of psychedelic-assisted psychotherapy. Firstly, there was European “psycholytic therapy” that was heavily based on psychoanalysis, i.e., Freudianism, that used small doses to *facilitate* the already-established psychoanalytic therapy (Passie, 1997; Majić et al., 2015, p. 245–246). Secondly, concurrently, in North America was practiced “psychedelic therapy” which, in contradistinction, used few but high doses of psychedelics (Grof, 2008 [1980], p. 21–47; Garcia-Romeu and Richards, 2018, p. 294). There were many other varieties of psychedelic-assisted therapy during these decades (ibid.), but these were the main strands. In addition to this therapeutic dichotomy, there is another: (i) psychedelic experience can assist psychotherapy, or (ii) psychotherapy can assist psychedelic experience and its effects. As psychiatrist Stanislav Grof—who conducted more than 4,000 psychedelic sessions (Grof, 2008 [1980], p. 13)—puts it:

“The first category involves approaches in which the emphasis is on systematic psychotherapeutic work; LSD is used to enhance the therapeutic process or to overcome resistances, blocks, and periods of stagnation. The approaches in the second category are characterized by a much greater emphasis on the specific aspects of the drug experience and the psychotherapy is used to prepare the subjects for the drug sessions, give them support during the experiences, and to help them integrate the material.” (Grof, 2008 [1980], p. 33)

After a couple of decades following prohibition, licences were granted to a few researchers, the most known of which is Rick Strassman's work on DMT between 1990 and 1995 (Strassman, 2001). Along with some others, this research was carried out *not* primarily for therapy but for understanding biological process correlated to the mind using “healthy normal volunteers” (Garcia-Romeu and Richards, 2018, p. 294). As the 21st century arrived, psychedelic research returned its gaze again to therapy.

The purpose of this text is primarily to enrich therapy. However, the Metaphysics Integration proposed below can also be implemented for those with no desire for therapy but rather for enrichment of life. Let us look at the current state of integration in psychedelic-assisted psychotherapy so to provide context and reasons for its development.

Integration is the third, final phase of psychedelic-assisted psychotherapy. The first is the preparatory phase where a rapport—or “therapeutic alliance” (Garcia-Romeu and Richards, 2018, p. 300)—is developed between the participant and the therapists [often two, of different sexes (ibid., p. 298)]. The second phase is the drug session itself, again supervised by the same therapists. The final phase, integration, “typically begins 1–2 days after the drug session in a follow-up meeting between patient and therapist(s) to discuss the patient's experience and reflect upon its content in more depth” (ibid., p. 299–300; see also Krupitsky and Grinenko, 1997, p. 167; Grof, 2008 [1980], p. 147–149). These integrative meetings can occur weekly, sometimes for a number of months—practices vary. Can we ask what precisely “integration” is here?

There is no precision to be found here. It is a practice in its infancy. A recent overview of psychedelic integration (Bathje et al., 2022) begins by acknowledging that there are “many definitions of psychedelic integration, and the term encompasses a range of practices and techniques. This seems to have led to confusion about what integration is” (ibid., p. 1). Nonetheless, the authors submit what they consider to be a synthesised definition. I quote all but the last clause of the definition (as it is metaphysically presumptuous)^19^:

“Integration is a process in which a person revisits and actively engages in making sense of, working through, translating, and processing the content of their psychedelic experience. Through intentional effort and supportive practices, this process allows one to gradually capture and incorporate the emergent lessons and insights into their lives...” (ibid., p. 4)

Bathje et al. go on to identify ten models of integration (ibid., p. 5–7), “based on Indigenous worldviews and practices, Transpersonal Psychology, Jungian Psychology, Acceptance and Commitment Therapy, Psychodynamic Psychology, Somatic Psychology, Nature Relatedness, Biopsychosocialspiritual Models, and Harm Reduction” (ibid., p. 5). We have not the scope to explore each model here, but note that none of them involve recourse to metaphysics, through which mystical experience can attain meaning.

What is distinctive about psychedelic-assisted psychotherapy is the prefix. As psychedelics can occasion metaphysical experiences, some of which appear to bestow the most therapeutic benefit (see below), it seems that an integrative approach that omits metaphysics as one *element* of a broader integrative phase will be an inadequate form of psychedelic-assisted psychotherapy. We have seen Hofmann (2009 [1979], p. 206) and Shanon (2010 [2002], p. 380) make this point, but Bathje et al. make it more explicitly:

“[T]hese models may not provide a broad enough framework for practitioners or journeyers [participants] to respond to this full range of psychedelic experiences. … We believe integration will be most complete when expanding to address the full range of experiences people have with psychedelic substances. … In attempting to work holistically, those assisting journeyers with integration will need the humility to recognise the limitations of their training and knowledge, and limitations of their cultural conditioning and worldview. A skilled range of collaborators and referral sources are likely to be valuable in facilitating the integration process.” (Bathje et al., 2022, p. 11).

Concurrent to the integration phase, there is some evidence of a phenomenon that follows certain psychedelic experiences, named the “afterglow” (Majić et al., 2015; Sampedro et al., 2017; Gorman et al., 2021, p. 11). In this period of a few weeks, one finds in the participant “heightened mood, psychological flexibility, openness” which “may enable patients to explore new behaviours and ways of thinking” (Gorman et al., 2021, p. 11). Such an afterglow phase running parallel to the integration phase would naturally be conducive to the intelligible presentation and integration of various metaphysical positions potentially relevant to a participant who had a corresponding metaphysical experience.

Of more therapeutic effect than the afterglow phase is the *type* of experience that the participant undergoes. As Ko et al. (2022, p. 10) conclude in their overview: “[The] presence and intensity of the mystical psychedelic experience contributes to therapeutic efficacy, to include both symptom reduction and improved quality of life. This was clearly indicated in the studies reviewed, in forms of correlation, prediction, and/or mediation.” (see also especially Roseman et al., 2018; Mollaahmetoglu et al., 2021; Rothberg et al., 2021; Yaden and Griffiths, 2021; McCulloch et al., 2022). It is “mystical psychedelic experience” that has been shown to have the greatest therapeutic effects in psychedelic trials. The meaning of “mystical psychedelic experience” is always, as the term suggests, a little mysterious. Ko et al., refer to items on (the aforementioned) questionnaires that refer back especially to Stace's (1960) criteria, outlined above [in turn based on James' (1902) criteria and thought]. Walter Pahnke was the first to develop Stace's criteria into a questionnaire (the MEQ) and a study which involved questions relating to the following factors that comprise mystical states (using Pahnke's own words): Transcendence of Time and Space (loss of usual sense of time or of space), Positive Mood (joy, love, peace, or blessedness), Sense of Sacredness, Unity (Internal and External), Transiency of Unity, Objectivity, or Reality (insights into being and existence in general), Paradoxicality, and Alleged Ineffability (Pahnke, 1963, p. 283–296). Ralph Hood's M-Scale (1975) is also based on Stace, 1960, and therefore rather similar to Pahnke's scale (though it omits paradoxicality). Of these factors, all but ineffability are part of experiential metaphysics (ineffability is not really an experience, but rather an expression of the inability to report an experience). Time and space are core phenomena studied in Metaphysics that especially relate to the positions of Idealism, Neutral Monism, and the Transcendent. The transcendental idealist Immanuel Kant, for instance, argued that time and space were not real but merely projections of our mind (Kant, 2000 [1781/7], p. 155–192, A19/B33–A49/B73). The feeling of the unity of one's mind into the mind of Nature, along with joy and blessedness, finds a place of comprehension in Spinoza's Neutral Monism and Pantheism, through the concept of “the intellectual love of God/Nature” (Spinoza, 1985/1988, p. 609–617, *Ethics*, VP25–P42). Unity in terms of nature-connectedness or -exaltation can be framed within the panpsychological metaphysics of thinkers such as Alfred North Whitehead (e.g., Whitehead, 1958 [1938]; Gibson, 2020; Buchanan, 2022; Segall, 2022). The notion of “Objectivity or Reality” (related to the “noetic quality” of James, 1977 [1902]) refers to an intuition that the alternate reality one experiences is veridical rather than delusional (Pahnke, 1963, p. 290–291), which relates, for instance, to metaphysics vis-à-vis “possible worlds” (Lewis, 1986). Moreover, Pahnke's definition of “Objectivity or Reality” as “insights into being or existence in general” (ibid.) takes us back to Aristotle's original remit of metaphysics: the exploration of being *qua* being. We have seen further examples above of psychedelic-induced metaphysical experiences. For our purposes, what is important is that such experiences are regarded as therapeutically most effective, and that they are “mystical” experiences that can be more broadly reclassified as *metaphysical* experiences (see Figure 3). This reclassification assimilates them into a discipline that can foster a more meaningful and significant relationship between participants and their experiences.^20^

## Conclusion: The proposal and conjecture

Let us now bring together all that has been said to make the case for Metaphysics Integration as a part of psychedelic-assisted psychotherapy.

1. The psychedelic experiences which appear to have most (but not exclusive) therapeutic efficacy are metaphysical experiences. (Roseman et al., 2018; Mollaahmetoglu et al., 2021; Rothberg et al., 2021; Yaden and Griffiths, 2021; Ko et al., 2022; McCulloch et al., 2022).
2. There is evidence of an afterglow period after such experiences in which participants are open to new ideas about themselves and reality (Majić et al., 2015; Sampedro et al., 2017; Gorman et al., 2021, p. 11).
3. This afterglow period is concurrent to the Integration phase of psychedelic-assisted psychotherapy.
4. Psychedelic-assisted psychotherapy has been the province of psychologists, psychiatrists, and other counsellors who, as such, have not been trained in metaphysics.
5. Introducing an additional and optional Metaphysics Integration element into Psychedelic-assisted psychotherapy can be a more effective means of integrating metaphysical experience into a participant's sense of themselves and the reality in which they exist.
6. It is *conjectured* that this proposal, of Metaphysics Integration for psychedelic-induced metaphysical experiences, will produce *longer-term benefits* to participants. This is because (i) the experience can be more comprehensively framed, (ii) there will be less reason to dismiss the experience as delusional once a participant realises that each metaphysical position has an established legacy of discourse, (iii) relatedly, that the worldview hitherto adopted by the participant is but one metaphysical position amongst others, and (iv) that Metaphysics Integration *amplifies* the significance of the psychedelic experience that is regarded (point 1) as having the most therapeutic efficacy.

This is the proposal and the conjecture. There are many issues and questions that stem therefrom, as well as a number of potential projects and studies. One of the immediate issues is practical: how might Metaphysics Integration be implemented? This is an issue for another paper that considers how the complexity of metaphysics could be made intelligible to participants and practitioners through resources such as a handbook or practitioner training. The Metaphysics Matrix and its accompanying MMQ, provided here, can be used as the basis of such resources—and they show the possibility of simplification for intelligibility and practical use. Furthermore, we have seen throughout this text how certain psychedelic experiences can be discussed and made meaningful in terms of metaphysical positions. In practice this would need to be further bridged by the therapist to the participant's life, concerns, values, aims, and outlook. Since the 1950s there have been many varieties of psychedelic therapy, and there is no reason why Metaphysics Integration should not be an augmentation of such therapies currently in development. In fact, as I have sought to show, Metaphysics Integration may offer an advance in psychedelic-assisted psychotherapy—because integrating metaphysical experience requires recourse to metaphysics.

## Data availability statement

The original contributions presented in the study are included in the article/supplementary material, further inquiries can be directed to the corresponding author.

## Author contributions

The author confirms being the sole contributor of this work and has approved it for publication.

## Footnotes

^1^The other two are epistemology (theory of knowledge) and axiology (ethics and aesthetics).

^2^Osmond wrote here that psychedelic meant “mind-manifesting” (Osmond, 1957, p. 429). Though this was the first published use of the word, Osmond had proposed the word a year earlier in his correspondence with Aldous Huxley (see Dickins, 2022).

^3^See Hauskeller et al. (2022), for an overview and analysis of the relations between philosophy and psychedelics.

^4^The MMQ is already being used in one such research project: https://blogs.exeter.ac.uk/philosophyandpsychedelics/affiliates/projects/metaphysics-matrix-project/.

^5^This was said in an interview about logical positivism with Bryan Magee, on the BBC television show, *Men of Ideas*.

^6^The reference is to Ayer's posthumous collection of essays, but the original article, “What I Saw When I was Dead” was published in *The Sunday Telegraph* (28th August 1988).

^7^If we take the volume edited by Kim and Sosa (1999) as an exemplar of analytic metaphysics, we can learn what the subject concerns by looking at the sections of the volume: existence, identity, modalities and possible worlds, universals, properties, kinds, things and their persistence, the persistence of self, causation, emergence, reduction, supervenience, and realism/antirealism.

^8^Extract from “Lines Composed a few miles above Tintern Abbey, On Revisiting the Banks of the Wye during a Tour. July 13, 1798.” Wordsworth at this time was inspired by the Pantheism of Spinoza. Bullett (1950) classifies Wordsworth as an English mystic.

^9^Schrödinger expresses sympathies with biopsychism, Spinoza's Neutral Monism and Cosmopsychism, and a Transcendental Idealism (inspired by Kant, Schopenhauer, and Vedanta) (Schrödinger, 2008 [1961/4]).

^10^In Heisenberg's book *Physics and Philosophy* (Heisenberg, 2000 [1958]) he expresses much attraction to Kant's system of Transcendental Idealism.

^11^I add this process qualification because it is the qualm of process philosophy, sired by Whitehead (1861–1947), that the fundamental reality is not substance but process alone (see Sjöstedt-Hughes, 2021, p. 87–97).

^12^I claim this against those who believe that metaphysical reality is obviously physical (e.g., Sanders and Zijlmans, 2021). The so-called “comforting delusion objection”' to psychedelic-assisted psychotherapy (Pollan, 2015; Letheby, 2021) that claims that therapy is unethical if it does not abide by one particular metaphysical position—namely, Physicalism—may hinder therapy and may hinder it on the basis of an erroneous metaphysics—and ethics: with Stuart Hampshire, one might argue that “Ethics without metaphysics must be non-sense” (Hampshire, 1951, p. 115). An ethical reprimand against metaphysics would be seen as a reprimand against ethics' very condition of existence.

^13^This page range covers the sixth chapter named “Mystic Materialism.” The case of Honza Samotar therein (p. 214–220) is especially noteworthy.

^14^See Ganeri (2022) for the Indian influence upon Carpenter's notion of the term. But note, in addition to Ganeri's insightful analysis, that another influence of the name and concept was most likely the Hegelian term ‘eternal consciousness' that was popular in the Anglo-American Absolute Idealist circles of the time. Note also that psychiatrist R. M. Bucke named his 1901 book *Cosmic Consciousness* and took this state of mind to be an evolutionary development (Bucke, 1947 [1947 [1901]]).

^15^“One of the most convincing aspects of the mystic illumination is the apparent revelation of the oneness of all things, giving rise to pantheism in religion and to monism in philosophy” (Russell, 1951 [1914], p. 18).

^16^Because Otto defines this experience in terms not of unity but in terms of “The Wholly Other,” some commentators have sought to distinguish such experience as “numinous” in contrast to “mystical” (see Spilka et al., 2003, p. 292).

^17^“These are not escapes from but enlargements, burgeonings of reality. In so far as I can judge they occur in violation of Hughlings Jackson's principle, because the brain, although its functioning is impaired, acts more subtly and complexly than when it is normal. Yet surely, when poisoned, the brain's actions should be less complex, rather than more so?” (Osmond, 1957, p. 428) One might suspect the influence of Osmond's friend and associate Aldous Huxley (referenced in the 1957 paper) and his view that the brain is not productive but extractive of mind (Huxley, 2004 [1954/1956], p. 10).

^18^A “psychedelic-assisted logotherapy” whereby finding or giving a meaning to one's life is key to therapy (based on Frankl, 1969 [1946]) but aided *via* psychedelic experience, could be a worthwhile endeavour.

^19^The final clause reads, “… thus moving toward greater balance and wholeness, both internally (mind, body, and spirit) and externally (lifestyle, social relations, and the natural world).” (ibid.) The problem here is that there is a metaphysical presumption of a differentiation of “mind, body, and spirit,” as well as other implicit assumptions regarding nature, etc. It is such questions that psychedelic experience elicits; thus, it should not be imposed from the start thereby restricting avenues of thought.

^20^The sense of a grander scheme of reality that may emerge from psychedelic-induced metaphysical experiences may make participants' everyday problems appear in a new light of *relative insignificance*, thereby lessening the problems' emotional impact, and in turn lessening their need to be masked by alcohol abuse, etc. To speculate, this may be one reason for the therapeutic efficacy of such metaphysical experiences.

## Appendix 1:

**Metaphysics Matrix Questionnaire (MMQ)/Glossary**.

Based on the *Metaphysics Matrix* (Figure 1).

This pre-validated MMQ has been developed by Peter Sjöstedt-Hughes and Joseph Rennie

Answers: 1–7 (1. Strongly Agree 2. Agree 3. Somewhat Agree 4. Neutral 5. Somewhat Disagree 6. Disagree 7. Strongly Disagree)

**Physicalism**

1. Everything is fundamentally physical. [physicalism generally]
2. There is no such thing as the mental. [eliminativism]
3. What is meant by ‘mental' is only a person's behaviour. [logical behaviourism]
4. Mental activity is identical to patterns of brain activity. [psychoneural identity theory]
5. The mental is a functional network that as such can be realised by the brain but also by a machine. [functionalism]
6. Mental activity is not the same thing as brain activity but emerges from brain activity. [emergentism].
7. Mental activity *emerges* from brain activity and has power to alter the brain and body *via* desires, thoughts, and so on. [emergentism + mental causation]
8. Mental activity emerges from brain activity but has no power to alter the brain and body [epiphenomenalism]
9. Everything is fundamentally physical, but the physical always has a mental element. [real naturalism]
10. Everything is fundamentally physical and there are no gods. [physicalist atheism]

**Idealism**

11. Only my mind exists. [solipsism]
12. The reality we perceive is a projection of our minds. [idealism generally]
13. Every entity has a mind and reality appears differently to each such entity. [monadic idealism]

**Dualism**

14. The soul is distinct from the body. [substance dualism]
15. The soul is distinct from the body, yet they interact. [interactionism—non-interactionism (occasionalism, pre-established harmony)]
16. Everything has a soul. [animism]

17. The soul is infinite before and after birth. [infinite dualism]
18. The soul reincarnates. [metempsychosis]

**Neutral monism**

19. The physical and the mental are two aspects of the same fundamental substance. [neutral monism generally]
20. When we perceive something, it becomes part of us. [organic realism]
21. All entities have their own mental activity, from complex organisms to subatomic particles. [panpsychism]
22. All organisms, from humans to bacteria, have their own mental activity. [biopsychism]
23. All animals have their own mental activity. [zopsychism]
24. Stars and planets have their own mental activity. [astropsychism, polytheism]
25. The universe as a whole has its own mental activity. [cosmopsychism and pantheism]
26. My self extends beyond my brain, body, into the surroundings. [extended mind theory]

**The transcendent**

27. There exist conscious entities beyond ordinary human perception. [transcendent entities: demons, angels, ghosts, DMT entities, etc.]
28. There exists a realm outside time and space. [transcendent realm]
29. There exist more than three dimensions of space. [hyperspace]
30. The future already exists. [fatalism]
31. The universe has a purpose. [cosmic teleology]
32. Good and Evil are fundamental aspects of reality. [moral realism—nihilism]
33. Good alone is a fundamental aspect of reality. [moral realism]

**Theism**

34. There are gods. [polytheism]
35. There is a god. [monotheism]
36. God is the universe. [pantheism]
37. God is the universe and more. [panentheism]
38. God created the universe but then left it to itself to develop. [deism]
39. God is evil. [dystheism]
40. I hold no beliefs/Knowledge. [agnosticism]
